# Evaluation of the Atellica COAG 360 coagulation analyzer in a specialized coagulation laboratory

**DOI:** 10.1002/jcla.24276

**Published:** 2022-02-11

**Authors:** Karin Strandberg, Cecilia Augustsson

**Affiliations:** ^1^ Division of Laboratory Medicine, Coagulation Department of Clinical Chemistry and Pharmacology University and Regional Laboratories Region Skåne Malmö Sweden

**Keywords:** Atellica COAG 360, automated analyzer, coagulation, hemophilia, hemostasis

## Abstract

**Background:**

Diagnosis of bleeding disorders includes correct analysis of coagulation factors VIII, IX, XI, XII, XIII, II, V, VII, and X and von Willebrand antigen and activity. The aim of this study was to evaluate the analytical performance of the Atellica COAG 360 analyzer in a specialized coagulation laboratory with focus on specific coagulation parameters involved in the diagnosis of bleeding disorders.

**Methods:**

Verification included assessment of precision, reference interval, and method comparison according to local guidelines. For FVIII (Chromogenix) and FIX (Rossix), extended verifications were performed with additional assessment of linearity, detection limit, and comparability to BCS‐XP.

**Results:**

The precision was below 5% (normal levels) and below 10% (abnormal levels) and either improved or similar when compared to expected target values from a BCS‐XP. The locally established reference range agreed well (≥80% of measured values within manufacturer's assigned ranges) for most of the methods. The lower limit of quantification was calculated to below 0.01 IU/ml for FVIII chromogenic (Chromogenix) and FIX chromogenic (Rossix), both with acceptable linearity. Bland–Altman analyses revealed generally good agreement between Atellica COAG 360 and BCS‐XP in the determination of coagulation parameters, and differences between the two instruments did not result in any diagnostic change.

**Conclusions:**

The results of the evaluation show that the Atellica COAG 360 analyzer performs as expected to target values and equivalent to BCS‐XP for the diagnosis of bleeding disorders in a specialized coagulation laboratory providing service to a hemophilia treatment center (HTC).

## Graphical abstract

In this correlation study method comparisons were performed using Bland–Altman plots. Difference was calculated as the difference in results obtained on the Atellica COAG 360 analyzer and BCS‐XP for several analyses involved in the diagnosis of bleeding disorders.

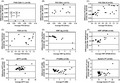

## 1 INTRODUCTION

Diagnosis of bleeding disorders in a specialized coagulation laboratory involves the correct diagnosis of hemophilia A, B, and C, von Willebrand disease, factor XIII (FXIII), factor XII (FXII), factor II (FII), factor V (FV), factor VII (FVII), and factor X (FX) deficiency. Hemophilia A, B, and C are characterized by factor deficiency of factor VIII (FVIII), factor IX (FIX), and factor XI (FXI), respectively. Whereas FXII deficiency is of great importance for explaining a prolonged clot time, although a deficiency is not correlated with any bleeding, deficiencies of FII, FV, FVII, FX, and FXIII give an increased bleeding tendency.^1^ Correct diagnosis of von Willebrand disease involves many different methods and this study will only focus on the methods that can be performed on an automated analyzer such as von Willebrand factor (VWF) activity, based on the glycoprotein Ib‐containing gain‐of‐function mutation (VWF:GPIbM), and antigen (VWF:Ag). The recommendation for laboratories that perform diagnosis of hemophilia A and B is to have two methods available for factor activity measurements covering both one‐stage assay (OSA) and chromogenic substrate assay (CSA).^2^, ^3^, ^4^ In addition, the need to distinguish severe from moderate hemophilia requires a method with low detection limit, below 0.01 IU/ml factor activity.^3^ The need for CSA and OSA for factor activity measurement of FVIII and FIX is also demonstrated by assay discrepancy seen for extended half‐life (EHL) products, which was covered previously.^5^, ^6^, ^7^, ^8^ While high‐throughput and short turnaround times are essential requirements for coagulation analyzers in routine laboratories, the requirements for specialized laboratories that provide services to a hemophilia treatment center (HTC) are somewhat different. Besides correct phenotype and severity classification for hereditary bleeding disorders, testing also involves diagnosing acquired or treatment‐induced bleeding conditions, some of which are considered critical hemostasis tests (i.e., anti‐FXa activity for heparin/low molecular weight heparin [LMWH]).^9^

The Atellica COAG 360 analyzer from Siemens Healthineers is a fully automated hemostasis analyzer. Besides performing coagulation testing using different techniques such as clotting, immunologic assay, luminescent oxygen channeling (LOCI) assay, and aggregation testing, it features a HIL check (hemolysis, icterus, and lipemia) and storage of reagents in a cooled compartment. Hörber and colleagues evaluated the Atellica COAG 360 analyzer in a central laboratory and concluded that it provided high analytical performance.^10^ The performance of OSA and CSA methods has also been tested for hemophilia replacement therapy with different EHL products using Atellica COAG 360.^5^, ^11^

The aim of the present study was to evaluate the analytical performance of the Atellica COAG 360 analyzer in a specialized coagulation laboratory with focus on specific coagulation parameters involved in the diagnosis of hereditary and acquired bleeding disorders. The methods provided by Siemens on Atellica COAG 360, included in this study, covers chromogenic assays for FVIII, FXIII, and anti‐FXa activity (LMWH); OSA for FVIII, FIX, FXI, FXII, FII, FV, FVII, and FX; immunoturbidimetric assay for VWF:Ag; and latex‐based VWF platelet‐binding assay (in the absence of ristocetin) for VWF:GPIbM.^12^ Methods not provided by Siemens, also adopted on the Atellica COAG 360 analyzer, were chromogenic assays for FVIII and FIX using third‐party reagents that required laboratory developed test (LDT) and an extended verification.

Verification of precision on at least two levels of control material and reference range was performed for all methods. Comparability to the previous BCS‐XP method, linearity and detection limit were performed on selected methods in accordance with international guidelines and local regulatory requirements.^13^

## 2 MATERIALS AND METHODS

### 2.1 Sample collection

This was a method comparison in which samples from routine clinical follow‐up and spiked normal plasma were analyzed in coagulation assays for comparability studies. Ethical approval was obtained from the local Ethics Board (Dnr2015/886). Normal pooled platelet poor plasma was obtained in house from male (26%) and female donors (74%) (*n* = 50, aged 22–70) with informed consent. Blood samples (3.2% sodium citrate, 109 mmol/L) were collected and centrifuged for 20 min at 2000 *g* at room temperature. Plasma supernatants were frozen and stored at −70°C until analysis.

### 2.2 Study design

Verification included assessment of precision, reference interval, and method comparison according to local guidelines from the Swedish national accreditation body, SWEDAC, using Swedac DOC 01:55, 2011‐08*10 release 4 for ISO15189 accreditation and when applicable to target values obtained for the previous BCS‐XP method.^14^ For FVIII and FIX chromogenic assays that required a LDT, an extended verification was performed with the above verification requirements and additional assessment of linearity and detection limit according to guidelines.^3^ Comparability was assessed to BCS‐XP for the analyses in the screening panel for bleeding disorders (APTT, PT(INR), Quick's PT, FVIII CSA‐1, FIX CSA, and VWF:GP1bM) with addition of VWF:Ag, FXIII, and anti‐FXa activity (LMWH), which was required by local guidelines when not provided by the manufacturer for the reagent and instrument combination.

### 2.3 Reagents, calibrators, and controls

Reagents and calibrators were used on the Atellica COAG 360 analyzer (Siemens Healthineers) according to the instructions provided by the manufacturer (Table 1). Factor VIII chromogenic activity was measured using two different CSA methods; CSA‐1 (see below) and CSA‐2 (Siemens). Methods not provided by Siemens and thus required LDT were chromogenic assays for FVIII (CSA‐1, Chromogenix, IL Company) and FIX (Rossix), with normal reference plasma (NRP) from Precision Biologic as calibrator. Calibration included a high (calibration points ≥ 0.125 IU/ml, for samples > 0.20 IU/ml) and a low (calibration points ≤ 0.25 IU/ml, for samples ≤ 0.20 IU/ml) calibration curves using at least 5 points per curve. Reagent for prothrombin time using Owren was from Medirox with the national INR calibrator from Equalis. Controls were from Siemens, except for the low FIX controls (0.1 and <0.05 IU/ml), which were from Precision Biologic. The same reagents were used on the BCS‐XP (Siemens) except for STA‐PTT automate (Diagnostica Stago) and Thromborel S (Siemens) as OSA reagents and Coamatic heparin (Chromogenix) as anti‐FXa activity (LMWH) reagent.

**TABLE 1:** Reagents used on the Atellica COAG 360 analyzer

Method	Reagent	Calibrator	Factor‐deficient plasma
APTT	Actin FSL (Siemens Healthineers)	n.a	n.a
PT(INR)	Owren (Medirox)	INR calibrator (Equalis)	n.a
Quick's PT	Innovin^®^ (Siemens)	n.a	n.a
FVIII CSA−1	Coatest SP (Chromogenix)	NRP (Precision Biologic)	Immunodepleted (Siemens)
FVIII CSA−2	Chromogenic (Siemens)	SHP (Siemens)	Immunodepleted (Siemens)
FVIII OSA	Actin FS (Siemens)	SHP (Siemens)	Immunodepleted (Siemens)
FIX CSA	ROX Factor IX (Rossix)	NRP (Precision Biologic)	Congenital (George King Bio‐Medical)
FIX OSA	Actin FS (Siemens)	NRP (Precision Biologic)	Congenital (George King Bio‐Medical)
VWF:Ag	VWF Ag (Siemens)	SHP (Siemens)	n.a
VWF:GPIbM	INNOVANCE^®^ VWF Ac (Siemens)	SHP (Siemens)	n.a
FXI and FXII OSA	Actin FS (Siemens)	SHP (Siemens)	Immunodepleted (Siemens)
FXIII	Berichrom^®^ (Siemens)	SHP (Siemens)	n.a
FII, FV, FVII, and FX OSA	Innovin^®^ (Siemens)	SHP (Siemens)	Immunodepleted (Siemens)
Anti‐FXa activity (LMWH)	INNOVANCE^®^ Heparin (Siemens)	INNOVANCE^®^ Heparin calibrator (Siemens)	n.a

Abbreviations: Ag, antigen; APTT, activated partial thromboplastin time; CSA, chromogenic substrate assay; GPIbM, glycoprotein Ib‐containing gain‐of‐function mutation; INR, international normalized ratio; LMWH, low molecular weight heparin; n.a, not applicable; NRP, normal reference plasma; OSA, one‐stage assay; PT, prothrombin time; SHP, standard human plasma.

### 2.4 Assessment of linearity and detection limit

A linearity test was performed using manual dilution of the normal pooled plasma in FVIII‐deficient plasma for the FVIII CSA‐1 method and dilution of standard human plasma (Siemens) in FIX‐deficient plasma (George King Bio Medical) for the FIX CSA method. Samples were measured in duplicates and a comparison of the theoretical assigned value and measured value was evaluated using linear regression analysis, with *r* ^2^ as the coefficient of correlation. Linearity was assumed as acceptable when *r* ^2^ > 0.998.^3^ The detection limit for FVIII and FIX CSA assays was validated by measuring a blank sample, containing assay buffer, 20 times. Since most of the measurement would yield a result below the detection limit, the raw value (absorbance/min) was used in order to calculate the lower limit of detection (LLOD) as the mean + 3 SD. The lower limit of quantification (LLOQ) was calculated as three times the value of LLOD.^3^ A LLOQ < 0.01 IU/ml was considered acceptable for hemophilia severity classification.

### 2.5 Assessment of reference interval, precision, and comparability

Reference intervals were verified locally and performed by measuring the normal plasma pool individual donors from 30 to 50 individuals. For some parameters, ≥50 individuals were used and included both locally provided donors and purchased samples (Cryocheck normal donor set, Precision Biologic). A target value of ≥90% of measured values within manufacturer's assigned ranges was used.^15^ Reference range was reported as mean ± 2 standard deviations (SD) after normality test using D’Agostino and Pearson and Shapiro–Wilk tests (data not shown). Precision was determined by measuring control samples on a minimum of two levels per method, five times during 1 day over 5 days, yielding at least 25 measured results for each level and reported with a total coefficient of variation (CV%). At least two control levels were used for each calibration curve. A target CV% ≤ 5.0% on normal levels and ≤10.0% on abnormal levels were used.^16^ The target CV was chosen based on Marlar et al, i.e., the CV is usually accepted at 3%–6% for clotting, chromogenic, and most immunologic analytes but never more than 10%.^16^ Comparability was based on the performance on the previous BCS XP and performed on selected methods. Bland–Altman analysis was used for assessing bias. A bias <10% for 95% of samples was considered acceptable.^13^ Regression analysis was also performed, with a target of slope between 0.90 and 1.10 and Pearson *r* ^2^ ≥ 0.95 for the correlation assessment.^13^ A correlation study of anti‐FXa activity (LMWH) assay was conducted using the in‐house normal pooled plasma spiked with different amounts (1.0, 0.5, and 0.25 IU/ml) of Fragmin^®^ (Pfizer).

### 2.6 Statistical analysis

Figures, reference intervals, and comparisons were created and calculated using GraphPad Prism 8.0.2 (GraphPad Software). Linear regression (Pearson *r* ^2^) and Bland–Altman analyses were performed for comparison of coagulation measurement results.^17^

## 3 RESULTS

### 3.1 Assessment of linearity and detection limit

**FIGURE 1:**
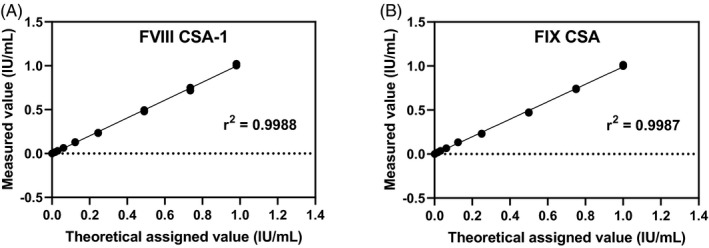
Assessment of linearity and detection limit. Normal pooled plasma was diluted in FVIII‐deficient plasma, and SHP in FIX‐deficient plasma and analyzed in duplicates in the relevant factor activity method. Measured values were plotted against the theoretical assigned values (IU/ml). Linear regression was made with the shown correlation coefficient (*r* ^2^). FVIII CSA‐1 (A) and FIX CSA (B). Abbreviation: CSA (chromogenic substrate assay)

For assays with reagents not provided by Siemens, i.e., CSA methods for FVIII and FIX, an assessment of linearity and detection limit were completed. Linearity was accepted with *r* ^2^ ≥ 0.998 for FVIII CSA‐1 and FIX CSA (Figure 1A,B). The detection limit, i.e., LLOQ, was calculated to <0.01 IU/ml (<1%) for FVIII CSA‐1 and FIX CSA, 0.004 and 0.009 IU/ml, respectively.

### 3.2 Reference interval

The reference interval was verified locally for all methods using 30–50 individual donors, Table 2. Good agreement was obtained for majority of methods (target: ≥90% of measured values within assigned ranges) when compared to manufacturer's assigned ranges, except for FIX CSA (76%), FVIII CSA‐2 (80%), FIX OSA (89%), FXII OSA (88%), FXIII (80%), FV OSA (83%), and Quick's PT (76%). For both FIX methods (OSA and CSA), an additional set of samples from healthy donors were included (total individual samples ≥ 50) in order to establish a reliable local reference interval. For FXII OSA, FV OSA, and FXIII, no further analysis was made due to low frequent used assays with limited patient numbers and medical consequences. The manufacturer's reference range for Quick's PT was 7.3–9.1 s, and thus covered within our locally obtained range (6–10 s, Table 2) and no further analysis was done. For PT(INR), the reference range (0.90–1.2 INR) from EQUALIS (National External Quality Organization) was used and verified locally (data not shown).

**TABLE 2:** Comparison of different coagulation assays on the Atellica COAG 360 analyzer used in the evaluation of bleeding disorders

Method	Reference interval			Comparability			
	Mean ±2 SD (n)		% within manufacturer's range	Bias from Bland–Altman	Linear regression, r^2^	Slope	N
APTT	21–30 s (49)		94	0.57 (1.7%)	0.97	1.01	39
PT(INR)	n.d			−0.06 (−4.0%)	0.99	1.01	39
Quick's PT	6–10 s (45)	76		−3.23 (−27.3%)	0.97	1.05	42
FVIII CSA−1	0.55–1.17 IU/ml (50)		100	−0.002 (−4.0%) L	0.97	0.84	19
FVIII CSA−2	0.64–2.09 IU/ml (54)		80	n.d			
FVIII OSA	0.69–1.93 IU/ml (30)		97	n.d			
FIX CSA	0.71–1.58 IU/ml (105)		76	0.003 (4.9%) L 0.040 (7.0%) H	0.98 0.99	1.04 1.07	11 33
FIX OSA	0.70–1.30 IU/ml (80)		89	n.d			
VWF Ag	0.58–1.65 IU/ml (50)		98	−0.15 (−5.2%)	0.99	0.77	23
VWF:GPIbM	0.47–1.81 IU/ml (50)		98	0.03 (1.6%)	0.99	1.06	24
FXI OSA	0.83–1.48 IU/ml (30)		96	n.d			
FXII OSA	0.65–1.70 IU/ml (30)		88	n.d			
FXIII	0.83–1.77 IU/ml (30)		80	0.02 (3.3%)	0.97	0.93	15
FII OSA	0.80–1.30 IU/ml (30)		93	n.d			
FV OSA	0.60–1.70 IU/ml (30)		83	n.d			
FVII OSA	0.60–1.60 IU/ml (30)		97	n.d			
FX OSA	0.70–1.40 IU/ml (30)		90	n.d			
Anti‐FXa activity (LMWH)	n.d			−0.02 (−1.2%)*	1.00	0.91	90

Note

Bias obtained by method comparison using the Bland–Altman analysis and calculated with the difference in results obtained on the Atellica COAG 360 analyzer and BCS‐XP. Linear regression analysis and the correlation (Pearson r^2^) were calculated. *Comparison in results on measured values from the Atellica COAG 360 analyzer and assigned value.

Abbreviations: Ag, antigen; and H, High factor levels, including above 0.10 IU/ml; APTT, activated partial thromboplastin time; CSA, chromogenic substrate assay; GPIbM, glycoprotein Ib‐containing gain‐of‐function mutation; INR, international normalized ratio; L, Low factor levels, below 0.10 IU/ml; LMWH, low molecular weight heparin; n.d, not determined; OSA, one‐stage assay; PT, prothrombin time; SD, standard deviation.

### 3.3 Precision

In general, the total coefficient of variation (CV) reached the assigned target values, below 5.0% for normal levels and below 10.0% for abnormal levels except for 10.1% at 0.05 IU/ml FVIII OSA and 5.6% at 0.9 IU/ml FXIII, see Table 3. For most of the methods, CVs were improved on the Atellica COAG 360 analyzer when compared to BCS‐XP. Methods with a large improvement consisted of one‐stage‐based factor assays.

**TABLE 3:** Precision of coagulation parameters determined by BCS‐XP and the Atellica COAG 360 analyzer

Parameter	BCS‐XP		Atellica COAG 360	
	Level	Total CV (%, *n* = 25)	Level	Total CV (%, *n* = 30)
APTT	64 s	1.8	74 s	1.9
	30 s	1.0	28 s	0.7
PT (INR)	2.7 INR	1.6	2.7 INR	5.0
	1.0 INR	1.0	1.0 INR	2.1
Quick's PT	*Thromborel S*		*Innovin*	
	21 s	4.3	16 s	1.7
	13 s	1.7	9.0 s	1.1
FVIII CSA−1	1.0 IU/ml	6.3	0.9 IU/ml	3.0
	0.3 IU/ml	9.2	0.3 IU/ml	5.8
	0.2 IU/ml	4.3	0.1 IU/ml	3.4
	0.06 IU/ml	7.3	0.06 IU/ml	4.5
FVIII CSA−2	n.d	n.d	0.8 IU/ml	3.2
	n.d	n.d	0.3 IU/ml	4.8
	n.d	n.d	0.06 IU/ml	4.7
FVIII OSA	*STA‐PTT automate*		*Actin FS*	
	0.8 IU/ml	10.0	1.0 IU/ml	4.4
	0.3 IU/ml	8.9	0.3 IU/ml	4.9
	0.05 IU/ml	16.9	0.06 IU/ml	10.1
FIX CSA	0.8 IU/ml	11.1	0.9 IU/ml	2.8
	0.3 IU/ml	7.9	0.3 IU/ml	2.2
	0.09 IU/ml	4.7	0.1 IU/ml	8.5
	0.02 IU/ml	7.8	0.03 IU/ml	5.6
FIX OSA	*STA‐PTT automate*		*Actin FS*	
	0.8 IU/ml	8.7	1.0 IU/ml	4.8
	0.3 IU/ml	12.1	0.4 IU/ml	5.7
	0.1 IU/ml	9.1	0.1 IU/ml	5.5
	0.03 IU/ml	6.4	0.02 IU/ml	8.7
VWF:Ag	1.3 IU/ml	2.7	1.2 IU/ml	2.1
	0.4 IU/ml	2.9	0.4 IU/ml	3.5
	0.1 IU/ml	4.6	0.1 IU/ml	3.8
VWF:GPIbM	1.0 IU/ml	4.8	1.0 IU/ml	1.5
	0.3 IU/ml	3.7	0.3 IU/ml	1.8
	0.1 IU/ml	2.9	0.1 IU/ml	1.6
FXI OSA	*STA‐PTT automate*		*Actin FS*	
	0.9 IU/ml	6.2	1.0 IU/ml	3.3
	0.3 IU/ml	9.6	0.4 IU/ml	6.1
FXII OSA	*STA‐PTT automate*		*Actin FS*	
	1.0 IU/ml	10.4	1.2 IU/ml	3.2
	0.3 IU/ml	14.1	0.3 IU/ml	2.6
FXIII	1.0 IU/ml	5.5	0.9 IU/ml	5.6
	0.3 IU/ml	6.7	0.3 IU/ml	8.6
FII OSA	*Thromborel S*		*Innovin*	
	1.0 IU/ml	9.5	0.9 IU/ml	2.2
	0.3 IU/ml	8.8	0.3 IU/ml	2.0
FV OSA	*Thromborel S*		*Innovin*	
	0.9 IU/ml	7.8	1.0 IU/ml	3.9
	0.3 IU/ml	9.0	0.3 IU/ml	4.2
FVII OSA	*Thromborel S*		*Innovin*	
	1.1 IU/ml	5.8	1.0 IU/ml	2.9
	0.4 IU/ml	6.9	0.4 IU/ml	3.0
FX OSA	*Thromborel S*		*Innovin*	
	1.0 IU/ml	6.5	0.9 IU/ml	3.9
	0.3 IU/ml	5.6	0.3 IU/ml	3.2
Anti‐FXa activity (LMWH)	*Chromogenix*		*INNOVANCE*	
	0.8 IU/ml	1.8	1.1 IU/ml	2.4
	0.4 IU/ml	10	0.4 IU/ml	2.7

Abbreviations: Ag, antigen; and LMWH, low molecular weight heparin; APTT, activated partial thromboplastin time; CSA, chromogenic substrate assay; GPIbM, glycoprotein Ib‐containing gain‐of‐function mutation; INR, international normalized ratio; n.d, not determined; OSA, one‐stage assay; PT, prothrombin time.

### 3.4 Accuracy and comparability study: Atellica COAG 360 vs. BCS‐XP

**FIGURE 2:**
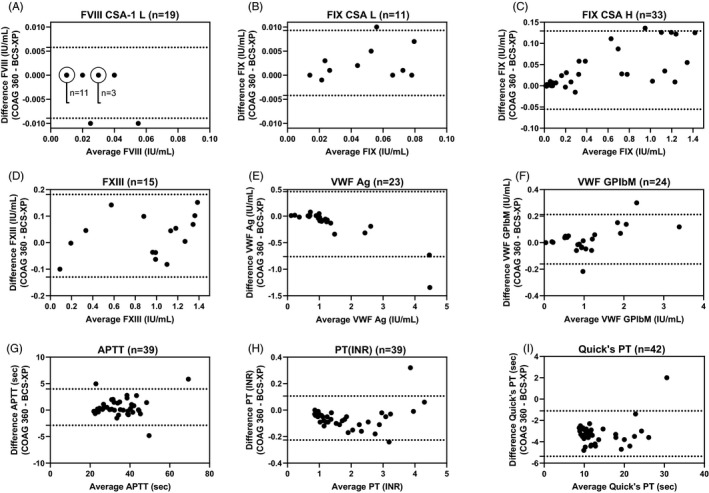
Correlation study. Method comparison using Bland–Altman. Difference was calculated as the difference in results obtained on the Atellica COAG 360 analyzer and BCS‐XP. Dotted lines represent the 95% limits of agreement computed as the mean difference (bias) plus or minus 1.96 times its standard deviation. (A) 19 samples at low factor levels below 0.10 IU/ml (L) were analyzed in FVIII activity using CSA‐1. (B) 11 samples at low factor levels below 0.10 IU/ml (L) were analyzed in FIX activity using CSA. (C) Additional 22 (total 33 samples) including factor levels above 0.10 IU/ml (H) were analyzed in FIX activity using CSA. (D) 15 samples were analyzed in FXIII activity. (E) 23 samples were analyzed in VWF:Ag. (F) 24 samples were analyzed in VWF:GPIbM. (G) 39 samples were analyzed in APTT clot time. (H) 39 samples were analyzed in PT (INR) clot time. (I) 42 samples were analyzed in Quick's PT. Abbreviations: CSA (chromogenic substrate assay), OSA (one‐stage assay), Ag (antigen), GPIbM (glycoprotein Ib‐containing gain‐of‐function mutation), APTT (activated partial thromboplastin time), PT (prothrombin time), and INR (international normalized ratio)

Correlation studies were made using the CSA methods, CSA‐1 (Chromogenix) for FVIII and CSA (Rossix) for FIX. Patient samples with low factor activity (below 0.10 IU/ml) were collected and analyzed on the two analyzers. Comparison of the results was evaluated using the Bland–Altman method and the results can be seen in Figure 2A,B and Table 2. At low factor levels below 0.10 IU/ml (L), a low bias was obtained (−0.0016; −4.0% and 0.0025; 4.9% for FVIII and FIX, respectively). The comparability study of FIX CSA also included patient samples at factor activity levels above 0.10 IU/ml (H), which also showed low bias (0.040; 7%), see Figure 2C and Table 2. For FVIII, FIX, FXI, and FXII OSA, correlation studies were not made since these OSA methods were performed according to the instrument and reagent manufacturer Siemens on Atellica and were LDTs on BCS‐XP (Actin FS on Atellica COAG 360 and PTT automate on BCS‐XP). Bland–Altman plots revealed good agreement between analyzers for the other parameters VWF:Ag, VWF:GPIbM, APTT, PT(INR), and FXIII (see Figure 2D–I and Table 2). Although one result with high discrepancy for VWF:Ag and one for VWF:GPIbM, these results were at values above 2 IU/ml and thus did not change the diagnosis of the patients (Figure 2E,F). Correlation studies on Quick's PT revealed good correlation (*r* ^2^ > 0.95, Table 2) and Bland–Altman plots revealed relative high bias (−3.23; 27%, Table 2). For FII, FV, FVII, and FX OSA, a correlation study was not performed due to lack of patient samples over the measuring range. However, for the anti‐FXa activity (LMWH) assay, spiked samples showed acceptable agreement with low bias (−0.02; −1.2%) when compared to the assigned value (Table 2).

Regression analysis revealed optimal comparability (slope 0.90–1.10) for the majority of the assays and suboptimal comparability for FVIII CSA‐1 and VWF:Ag (slopes at 0.84 and 0.77, respectively), see Table 2.

## 4 DISCUSSION

The Atellica COAG 360 analyzer was verified and validated as a new coagulation analyzer for use in our specialized laboratory for the diagnosis of bleeding disorders. Verification (precision, reference range, and accuracy) was performed on methods and reagents provided by Siemens, while an extended verification (including additional assessment of linearity and detection limits) was performed on methods and reagents provided by a third party, i.e., chromogenic FVIII and FIX assays. To our knowledge this has not been reported previously for the Atellica COAG 360 analyzer. A similar verification was made for all methods comprising our evaluation of thrombosis disorders, although this was not within the scope of this study.

The precision was, in general, very good and comparable to results published by Hörber et al. with total CV below 10% for all methods at all levels, except for 10.1% measured at 0.05 IU/ml FVIII OSA. A slightly higher CV was obtained for OSA at the lowest factor level for both FVIII and FIX (0.05 IU/ml FVIII or 0.02 IU/ml FIX) compared to the CV at the other levels. The precision was either improved or quite similar for the Atellica COAG 360 analyzer when compared to precision obtained using BCS‐XP. The improved precision seen using the Atellica COAG 360 analyzer mainly applied to the methods using OSA reagents. It is difficult to conclude whether this improvement occurred because of the new analyzer or was due to the fact that different OSA reagents were used on the BCS‐XP (PTT‐automate) compared to the Atellica COAG 360 analyzer (Actin FS), with assay protocol differences. Our laboratory participates in the ECAT EQA scheme and performance has been acceptable/comparable to BCS‐XP, except for Quick's PT on normal values which can be explained by a lower reference range on the Atellica COAG 360 analyzer.

The linearity and detection limits of the chromogenic assays (FVIII and FIX LDTs) were all approved. Unfortunately, the chromogenic assay provided by Siemens (here stated as CSA‐2) has a reported detection limit of 0.035 IU/ml that was also confirmed in our setting (0.024 IU/ml data not shown) which did not meet our requirement for the detection limit, <0.01 IU/ml.^12^ As a consequence, in our laboratory that provides service to a HTC, we need to use a LDT method with third‐party reagent (CSA‐1) for chromogenic FVIII activity measurements in order to differentiate between the moderate form and severe form.^18^ Improvements of the CSA‐2 assay were not investigated (e.g., addition of a lower calibration point by dilution of calibrator and prolonged incubation time) and would result in a LDT method. However, the CSA‐2 could be useful when assaying post‐infusion samples of patients given EHL products.^5^, ^19^

The reference range was verified locally for all parameters in the bleeding panel. Clot times in APTT and Quick's PT were markedly shorter using the Atellica COAG 360 analyzer but in line with the manufacturer's reported reference range and, therefore, the reference interval diverged from that using the BCS‐XP. For Quick's PT, the difference in reference interval and high bias when compared to BCS‐XP might be explained by the different reagents used, Innovin on Atellica COAG 360 while Thromborel S on BCS‐XP. The level of VWF antigen and activity and thus activity of FVIII are known to be slightly decreased for patients with blood group 0.^20^ This was not taken into consideration when establishing the reference intervals, and blood group‐independent reference intervals are shown in this report.

Correlations studies revealed good correlation for the majority of the assays analyzed. The high bias of 27.3% for Quick's PT was in line with the locally obtained reference range, which was 20%–40% lower on Atellica COAG 360 compared to BCS‐XP (data not shown). In addition, regression analysis distinguished FVIII CSA‐1 and VWF:Ag with suboptimal slopes below 0.90. This can be explained by the two patient samples with high discrepancy (Figure 2A,E), although no change in the diagnosis of the patients and therefore no additional actions were made.

Although the Atellica COAG 360 analyzer covers five different assay technologies, we have only investigated the clotting (optical detection), immunologic, and chromogenic assays. Additional features including HIL check, cooled storage of reagents, and aliquot sampling were not evaluated. In conclusion, the results of the evaluation show that the Atellica COAG 360 analyzer performs as expected to target values as an analyzer in a specialized laboratory with the methods and reagents tested for the diagnosis of bleeding disorders. Most importantly, the small differences between the compared instruments did not result in any diagnostic change for the patients.

## CONFLICT OF INTEREST

The authors state that there are no conflicts of interest.

## Data Availability

Available upon request.
